# Comparison of Sodium-Glucose Cotransporter 2 Inhibitors vs Glucagonlike Peptide-1 Receptor Agonists and Incidence of Dry Eye Disease in Patients With Type 2 Diabetes in Taiwan

**DOI:** 10.1001/jamanetworkopen.2022.32584

**Published:** 2022-09-22

**Authors:** Yu-Chen Su, Jia-Horung Hung, Kai-Cheng Chang, Chi-Chin Sun, Yi-Hsun Huang, Chaw-Ning Lee, Ming-Jui Hung, Chi-Chun Lai, Shih-Chieh Shao, Edward Chia-Cheng Lai

**Affiliations:** 1 Department of Ophthalmology, National Cheng Kung University Hospital, College of Medicine, National Cheng Kung University, Tainan, Taiwan; 2 Institute of Clinical Medicine, College of Medicine, National Cheng Kung University, Tainan, Taiwan; 3 School of Pharmacy, Institute of Clinical Pharmacy and Pharmaceutical Sciences, College of Medicine, National Cheng Kung University, Tainan, Taiwan; 4 Department of Pharmacy, Linkou Chang Gung Memorial Hospital, Taoyuan, Taiwan; 5 Department of Ophthalmology, Keelung Chang Gung Memorial Hospital, Keelung, Taiwan; 6 College of Medicine, Chang Gung University, Taoyuan, Taiwan; 7 Department of Dermatology, National Cheng Kung University Hospital, College of Medicine, National Cheng Kung University, Tainan, Taiwan; 8 Division of Cardiology, Department of Internal Medicine, Chang Gung Memorial Hospital, Keelung, Taiwan; 9 Department of Pharmacy, Keelung Chang Gung Memorial Hospital, Keelung, Taiwan

## Abstract

**Importance:**

Sodium-glucose cotransporter 2 (SGLT2) inhibitors have been found to improve low-grade systemic and tissue inflammation; however, the association between SGLT2 inhibitor use and the incidence of dry eye disease (DED) has not been explored.

**Objective:**

To investigate the association between SGLT2 inhibitor use and dry eye disease in patients with type 2 diabetes (T2D).

**Design, Setting, and Participants:**

A retrospective cohort analysis of the largest multi-institutional electronic medical records database in Taiwan was conducted to identify patients with T2D newly receiving SGLT2 inhibitors or glucagonlike peptide-1 receptor agonists (GLP-1 RAs) from 2016 to 2018. Data analysis was performed from March 1 to May 31, 2022. Propensity scores with inverse probability of treatment weighting were generated to enable homogeneous comparisons between the 2 groups.

**Exposures:**

Treatment with SGLT2 inhibitors or GLP-1 RAs.

**Main Outcomes and Measures:**

Incident dry eye disease, which was defined by clinical diagnoses, plus the related drug prescription. Cox proportional hazards regression models were used to estimate hazard ratios with 95% CIs for the risk of DED.

**Results:**

A total of 10 038 and 1077 T2D patients newly receiving SGLT2 inhibitors (mean [SD] age, 59.5 [12.1] years; 5689 [56.7%] men) or GLP-1 RAs (mean [SD] age, 58.5 [41.2] years; 587 [54.5%] men), respectively, were included in the analysis. The incidence of DED was lower in patients newly receiving SGLT2 inhibitors (9.0 events per 1000 person-years) compared with those receiving GLP-1 RAs (11.5 events per 1000 person-years), yielding a hazard ratio of 0.78 (95% CI, 0.68-0.89). Subgroup analyses indicated that the lowered DED risks associated with SGLT2 inhibitors in patients with T2D were similar across different age, sex, blood glucose level, and kidney function groups. Results from the sensitivity analyses (including the propensity score–matching approach, on-treatment analyses, and different follow-up periods of 1, 2, and 3 years) were similar to the main analyses.

**Conclusions and Relevance:**

The findings of this study suggest that patients with T2D newly receiving SGLT2 inhibitors may have a lower risk for DED compared with those receiving GLP-1 RAs. Prospective studies are needed to analyze these results.

## Key Points

**Question:**

Is the use of sodium-glucose cotransporter 2 (SGLT2) inhibitors vs glucagonlike peptide-1 receptor agonists (GLP-1 RAs) associated with dry eye disease among adults with type 2 diabetes (T2D)?

**Findings:**

In this cohort study in Taiwan including 10 038 patients with T2D newly receiving SGLT2 inhibitors, there was a significant risk reduction in the incidence of dry eye diseases compared with 1077 propensity score–weighted patients with T2D newly receiving GLP-1 RAs. The lower dry eye disease incidence with use of SGLT2 inhibitors was consistent across different subgroup and sensitivity analyses.

**Meaning:**

The findings of this study suggest that patients with T2D newly receiving SGLT2 inhibitors may experience a lower risk for dry eye disease compared with those receiving GLP-1 RAs.

## Teaser

This cohort study compares sodium-glucose cotransporter 2 (SGLT2) inhibitors vs glucagonlike peptide-1 receptor agonists for dry eye disease in patients with type 2 diabetes in Taiwan.

## Introduction

The International Diabetes Federation estimates the global population of individuals with diabetes to be 463 million in 2019 and 700 million in 2045.^1^ Morbidity associated with diabetes includes macrovascular diseases (atherosclerosis) and microvascular diseases (neuropathy, nephropathy, and retinopathy).^2^ Although diabetic retinopathy is a well-known ocular complication due to chronic hyperglycemia,^3^ dry eye disease (DED) is another of the most frequently encountered conditions associated with type 2 diabetes (T2D). It has been reported that DED affects about one-fifth of patients with T2D and reduces the patients’ quality of life.^4,5^

The pathophysiologic process of DED depends on factors that include production of tear film components, the health of the ocular surface and eyelids, the presence of inflammatory mediators in the tear film, and hormonal abnormalities.^6,7^ Dry eye disease can be generally categorized into aqueous tear deficiency, evaporative DED, and often a combination of both types.^6^ Although aqueous tear deficiency is caused by inadequate tear volume, evaporative DED is largely attributed to an insufficient lipid layer leading to excessive evaporation of tear film. Tear film hyperosmolarity may stimulate epithelial cell damage and ocular surface inflammation, leading to further film instability and triggering a vicious cycle of DED.^6^ Because inflammation is an important component of this cycle,^8^ in addition to the first-line therapy (artificial tears and ointments), topical anti-inflammatory agents, such as cyclosporine, are prescribed to tackle the underlying inflammation in the eyes.^9^

Sodium-glucose cotransporter 2 (SGLT2) inhibitors, a novel drug class with glucose-lowering and other pleiotropic effects for T2D treatment,^10^ have been demonstrated to enhance systemic anti-inflammatory effects by increasing fat use,^11^ modulating macrophage inflammatory pathways,^11^ and inducing low-grade ketonemia,^12^ which partially explain the kidney and cardiac protective effects observed in clinical trials.^13,14^ Cohort studies in the clinical setting also report beneficial outcomes associated with SGLT2 inhibitors in treatment of ocular inflammatory diseases including diabetic retinopathy, diabetic macular edema, and glaucoma.^15,16,17^ These findings suggest the anti-inflammatory benefits from SGLT2 inhibitor treatment may extend to the retina and the optic nerve head. Because chronic inflammation also plays a major role in the pathophysiologic process of DED,^8^ the use of SGLT2 inhibitors could theoretically lower the incidence of DED.

Current clinical trials and observational studies mostly focus on the association of SGLT2 inhibitor treatment with macrovascular and microvascular complications and mortality in patients with T2D. The literature lacks evidence regarding the influence of SGLT2 inhibitors on DED; therefore, the present study aimed to evaluate the risk of DED after SGLT2 inhibitor treatment in patients with T2D. Because glucagonlike peptide-1 receptor agonists (GLP-1 RAs) and SGLT2 inhibitors are prescribed for patients with T2D presenting with similar comorbidities (eg, being at high risk of or having already established atherosclerotic cardiovascular and chronic kidney disease), we selected GLP-1 RAs as our comparators to address concerns over confounding by indication.^18^ We hypothesized that, compared with GLP-1 RAs, SGLT2 inhibitors were associated with lower incidence of DED, probably due to anti-inflammatory effects on ocular diseases.

## Methods

### Database Information

Chang Gung Memorial Hospitals is the largest medical system in Taiwan, comprising 7 hospitals located throughout Taiwan and covering a total of 10 050 beds and 8.2 million outpatient visits per year.^19,20^ The Chang Gung Research Database (CGRD) collects deidentified electronic medical records with diagnosis, procedures, medications, and laboratory information on patients in Chang Gung Memorial Hospitals. Diagnoses recorded in the CGRD are based on the *International Classification of Diseases, Ninth Revision, Clinical Modification* (*ICD-9-CM*) in the period prior to 2016 and *International Statistical Classification of Diseases, 10th Revision, Clinical Modification* (*ICD-10-CM*) thereafter.^20^

The CGRD has been proven to be an important data source to provide real-world evidence in previous comparison studies.^16,17,21,22,23^ The data representativeness and the validity of diagnostic codes in the CGRD have also been validated.^15,16,20,24,25,26,27^ This study protocol was approved, with waiver of informed consent, by the institutional review board of the Chang Gung Medical Foundation, and we followed the Strengthening the Reporting of Observational Studies in Epidemiology (STROBE) reporting guideline in the reporting of our results.

### Study Design and Participants

To minimize unmeasured confounding factors and ensure comparability between groups, we implemented the active-comparator and new-user design in this retrospective cohort study.^28^ Included were adults with T2D newly receiving SGLT2 inhibitors (empagliflozin, dapagliflozin, and canagliflozin) or GLP-1 RAs (liraglutide and dulaglutide) from January 1, 2016, to December 31, 2018. We chose GLP-1 RAs as the active comparator in this study because they share similar pleiotropic effects with SGLT2 inhibitors, such as cardiovascular and renal benefits for T2D treatment.^29^ We defined the first date of use of the SGLT2 inhibitor or GLP-1 RA as the index date and the year before the index date as the baseline period. To identify incident cases, we excluded patients with DED diagnoses during the baseline period. To ensure sufficient information for the evaluation of comorbidities and comedications, we excluded patients lacking other clinical visits at baseline and patients lacking baseline data on hemoglobin A_1c_ (HbA_1c_), estimated glomerular filtration rate (eGFR), and urine albumin-creatinine ratio (UACR) for the evaluation of their T2D status. Furthermore, we excluded patients with certain comorbidities at baseline, such as keratitis,^30,31^ Sjögren syndrome,^31^ history of corneal transplant,^31^ and conjunctivochalasis,^31^ because these diseases may substantially affect the tear films.^32^ These diseases were also defined as exclusion criteria in previous studies.^30,31^ In addition, we excluded patients with baseline eGFR levels less than 30 mL/min/1.73 m^2^ to adhere to the drug label recommendations for SGLT2 inhibitors.^33^

### Study Outcome

The primary outcome was the incidence of DED. To ensure the outcome validity, we identified DED by using the clinical diagnosis codes for DED (*ICD-9-CM*: 37515; *ICD-10-CM*: H0412) and records of prescriptions for dry eye medications identified by the Anatomical Therapeutic Chemical (ATC) codes of S01XA18 or S01XA20. Specifically, because the dry eye medications (including artificial tears and topical cyclosporine) were prescribed in accordance with Taiwan’s National Health Insurance reimbursement guidelines (eTable 1 in the Supplement), the details of eye examinations were included in the pathologic DED reports for clinical review for reimbursement purposes. Thus, the combination of coded diagnoses and prescription records for dry eye medications enhanced the validity of our outcome diagnoses. We followed up patients in the study from the index date to the occurrence of DED, last clinical visit, death, or the end of the database (December 31, 2021). Data analysis was conducted from March 1, 2022, to May 31, 2022.

### Covariates

First, we included baseline comorbidities related to the ophthalmologic conditions (ie, myopia, presbyopia, glaucoma, uveitis, conjunctivitis, pterygium, pinguecula, and blepharitis), diabetic complications (ie, diabetic retinopathy, neuropathy, and nephropathy), cardiovascular comorbidities (ie, coronary artery disease, ischemic stroke, peripheral artery disease, heart failure, hypertension, atrial fibrillation, and dyslipidemia), and other chronic diseases (ie, asthma, hyperthyroidism, hypothyroidism, liver disease, benign prostate hyperplasia, chronic obstructive pulmonary disease, arthritis, gout, rheumatoid diseases, migraine, sleep apnea, depression, schizophrenia, and rosacea). We also evaluated the overall disease burden using the Charlson Comorbidity Index composite scores. Second, we evaluated concomitant medications, including cardiovascular comedications, diabetic comedications, and other comedications possibly increasing the DED risks, such as oral antihistamines, antidepressants, antianxiety medications, oral corticosteroids, and hormone replacement therapy.^34,35^ Details of the *ICD-9-CM* and *ICD-10-CM* codes of comorbidities and the ATC codes of comedications are presented in eTable 2 and eTable 3 in the Supplement. In addition, we included the data on HbA_1c_, eGFR, and UACR levels to better understand the glycemic and kidney functions at baseline.

### Glycemic Control and Kidney Function Changes

To determine the possible mechanisms behind DED risks among different treatments, we further evaluated the HbA_1c_, eGFR, and UACR values based on the complete data analyses after 1-, 2-, and 3-year periods of SGLT2 inhibitor or GLP-1 RA use.

### Subgroup Analyses

We conducted subgroup analyses according to the baseline characteristics and laboratory data for each subgroup. The DED risks were reevaluated for groups of patients of different ages (≤60 or >60 years), sex (male or female), HbA_1c_ (≤7 or >7% [to convert to proportion of total hemoglobin, multiply by 0.01]), eGFR (≤90 or >90 mL/min/1.73 m^2^), and UACR (≤30 or >30 mg/g) levels.

### Sensitivity Analyses

We conducted sensitivity analyses to examine the robustness of our results. First, we alternatively used the 1:1 propensity score–matching method to mimic the conditions of randomized clinical trials by directly comparing outcomes between 2 different treatments within the propensity score–matched pairs.^36,37^ Second, we carried out on-treatment analysis to evaluate nonadherence or drug switching after the index date. In this approach, we censored patients exceeding 90 days without a prescription refill of index drugs and those switching to another index drug class during the follow-up period. Third, we used the inverse probability of censoring weighting method to examine the association between informative censoring due to treatment switching or discontinuation and the result estimates.^38^ Fourth, we censored patients developing DED less than 180 days after the index date to avoid outcome misclassification bias. Fifth, we compared the incidence of DED between SGLT2 inhibitors and GLP-1 RAs during different follow-up periods up to 3 years to evaluate the risks of DED during different follow-up periods.

### Falsification Outcome

Falsification outcomes are often used to assess the likelihood of false-positive results of primary study outcomes that may be attributed to unmeasured confounders or suboptimal internal validity.^39^ We considered incident vitreous floaters (*ICD-9-CM*: 37924, *ICD-10-CM*: H4339), a degenerative ocular disease,^40^ as a falsification outcome because the incidence may be similar for patients receiving SGLT2 inhibitors and those receiving GLP-1 RAs.

### Statistical Analysis

We calculated the mean (SD) for continuous variables and numbers and frequencies for categorical variables. Median (IQR) was also used for continuous variables where appropriate. To generate more homogeneous comparisons between the SGLT2 inhibitor and GLP-1 RA groups,^41,42^ we used propensity scores with inverse probability of treatment weighting (IPTW) and trimmed patients with the most extreme 5% of propensity score values for the main, subgroup, and sensitivity analyses. We adopted IPTW adjustment in this study because it could deliver average treatment effects in the whole study population that mimic the target of inference from randomized clinical trials.^43^ The propensity scores were based on logistic regressions that included the baseline covariates, such as sex, age, comorbidities, comedications, glycemic controls, and kidney function, as listed in Table 1. We used the absolute standardized mean differences with a cutoff value of 0.1 to assess the balance of baseline covariates between the 2 treatment groups after IPTW adjustment.^44^ Hazard ratios (HRs) and 95% CIs for DED risk were estimated by Cox proportional hazards regression models. We designated the GLP-1 RA new users as the reference group, and a 2-sided value of *P* < .05 was considered statistically significant. Statistical analyses were performed using SAS Enterprise Guide, version 7.1 (SAS Institute Inc).

**Table 1.** Baseline Characteristics Before and After IPTW With 5% Trimming

Characteristic	Before IPTW using propensity score with 5% trimming			After IPTW using propensity score with 5% trimming		
	No. (%)		ASMD^a^	No. (%)		ASMD^a^
	SGLT2 inhibitors (n = 11 022)	GLP-1 RAs (n = 1328)		SGLT2 inhibitors (n = 10 038)	GLP-1 RAs (n = 1077)	
Age, mean (SD), y	59.9 (11.6)	58.0 (13.3)	0.15	59.5 (12.1)	58.5 (41.2)	0.03
Male	6423 (58.3)	653 (49.2)	0.19	5689 (56.7)	587 (54.5)	<0.01
Female	4599 (41.7)	675 (50.8)	0.19	4349 (43.3)	489 (45.4)	<0.01
Laboratory data						
HbA_1c_, median % (IQR)	8.5 (7.6-9.6)	9.2 (8.4-10.3)	0.39	8.6 (7.7-9.7)	8.9 (8.0-9.8)	0.05
eGFR, median (IQR), mL/min/1.73 m^2^	88.9 (71.1-108.6)	84.1 (57.4-111.1)	0.11	89.1 (71.2-108.8)	90.9 (68.3-114.4)	0.02
UACR, median (IQR), mg/g	26.1 (9.6-118.1)	58.3 (14.5-301.2)	0.22	26.8 (9.9-121.7)	41.0 (11.8-172.2)	0.02
Ophthalmologic conditions						
Myopia	48 (0.4)	10 (0.8)	0.04	45 (0.5)	7 (0.6)	0.03
Presbyopia	48 (0.4)	6 (0.5)	<0.01	45 (0.5)	6 (0.5)	<0.01
Glaucoma	308 (2.8)	46 (3.5)	0.04	268 (2.7)	31 (2.9)	0.01
Uveitis	11 (0.1)	1 (0.1)	<0.01	9 (0.1)	1 (0.1)	0.01
Conjunctivitis	849 (7.7)	107 (8.1)	0.01	754 (7.5)	83 (7.7)	<0.01
Pterygium/pinguecula	13 (0.1)	0	0.05	0	0	NA
Blepharitis	80 (0.7)	13 (1.0)	0.03	72 (0.7)	9 (0.8)	0.01
No. of ophthalmologic outpatient visits, mean (SD)	0.5 (1.8)	0.7 (2.6)	0.1	0.5 (1.9)	0.5 (6.4)	0.02
Diabetic complications						
Retinopathy	985 (8.9)	167 (12.6)	0.12	894 (8.9)	104 (9.7)	0.03
Neuropathy	1130 (10.3)	174 (13.1)	0.09	1045 (10.4)	112 (10.4)	<0.01
Nephropathy	3655 (33.2)	508 (38.3)	0.11	3371 (33.6)	369 (34.3)	0.02
Cardiovascular comorbidities						
Coronary heart disease	2009 (18.2)	182 (13.7)	0.12	1654 (16.5)	168 (15.6)	0.02
Ischemic stroke	463 (4.2)	49 (3.7)	0.03	399 (4.0)	42 (3.9)	<0.01
Peripheral artery disease	165 (1.5)	28 (2.1)	0.05	144 (1.4)	12 (1.1)	0.03
Heart failure	547 (5.0)	44 (3.3)	0.08	383 (3.8)	37 (3.4)	0.02
Hypertension	7348 (66.7)	913 (68.8)	0.04	6682 (66.6)	715 (66.4)	<0.01
Atrial fibrillation	312 (2.8)	26 (2.0)	0.06	240 (2.4)	21 (2.0)	0.03
Dyslipidemia	8284 (75.2)	972 (73.2)	0.04	7513 (74.9)	805 (74.7)	<0.01
Other comorbidities						
Asthma	291 (2.6)	40 (3.0)	0.02	266 (2.7)	24 (2.3)	0.02
Hypothyroidism/hyperthyroidism	295 (2.7)	51 (3.8)	0.07	267 (2.7)	34 (3.2)	0.03
Liver disease	1970 (17.9)	238 (17.9)	<0.01	1798 (17.9)	177 (16.5)	0.04
Benign prostate hyperplasia	605 (5.5)	77 (5.8)	0.01	559 (5.6)	59 (5.5)	<0.01
Chronic obstructive pulmonary disease	299 (2.7)	32 (2.4)	0.02	249 (2.5)	17 (1.6)	0.06
Arthritis	14 (0.1)	4 (0.3)	0.04	11 (0.1)	2 (0.2)	0.01
Gout	859 (7.8)	115 (8.7)	0.03	793 (7.9)	74 (6.9)	0.04
Rheumatoid disease	46 (0.4)	6 (0.5)	<0.01	38 (0.4)	7 (0.6)	0.04
Migraine	48 (0.4)	9 (0.7)	0.03	43 (0.4)	3 (0.3)	0.03
Sleep apnea	98 (0.9)	15 (1.1)	0.02	91 (0.9)	10 (0.9)	<0.01
Depression	161 (1.5)	22 (1.7)	0.02	145 (1.4)	13 (1.2)	0.02
Schizophrenia	42 (0.4)	8 (0.6)	0.03	40 (0.4)	3 (0.2)	0.03
Rosacea	10 (0.1)	1 (0.1)	<0.01	8 (0.1)	0	0.04
CCI scores, mean (SD)	2.7 (1.5)	3.0 (1.7)	0.19	2.7 (1.6)	2.7 (5.1)	<0.01
Previous admission	1436 (13.0)	246 (18.5)	0.15	1246 (12.4)	131 (12.2)	<0.01
Cardiovascular comedication						
Antiplatelet	3567 (32.4)	417 (31.4)	0.02	3151 (31.4)	324 (30.1)	0.03
Calcium channel blocker	4258 (38.6)	559 (42.1)	0.07	3910 (39.0)	419 (38.9)	<0.01
β-Blocker	2654 (24.1)	278 (20.9)	0.08	2280 (22.7)	228 (21.2)	0.04
ACEI or ARB	6638 (60.2)	853 (64.2)	0.08	6062 (60.4)	646 (60.0)	<0.01
Diuretic	1129 (10.2)	153 (11.5)	0.04	966 (9.6)	103 (9.6)	<0.01
Statin	7526 (68.3)	895 (67.4)	0.02	6836 (68.1)	722 (67.1)	0.02
Fibrate	1078 (9.8)	149 (11.2)	0.05	1002 (10.0)	107 (9.9)	<0.01
Ezetimibe	1252 (11.4)	163 (12.3)	0.03	1141 (11.4)	133 (12.4)	0.03
Diabetes comedication						
Metformin	10 055 (91.2)	1097 (82.6)	0.26	9151 (91.2)	987 (91.7)	0.02
Sulfonylurea	6365 (57.8)	792 (59.6)	0.04	5979 (59.6)	603 (56.0)	0.07
Dipeptidyl peptidase-4 inhibitor	7301 (66.2)	959 (72.2)	0.13	6835 (68.1)	746 (69.3)	0.03
Thiazolidinedione	2902 (26.3)	257 (19.4)	0.17	2603 (25.9)	287 (26.6)	0.02
Acarbose	2013 (18.2)	293 (22.1)	0.09	1874 (18.7)	219 (20.4)	0.04
Glinide	245 (2.2)	69 (5.2)	0.16	211 (2.1)	24 (2.2)	<0.01
Insulin	2192 (19.9)	749 (56.4)	0.81	2090 (20.8)	242 (22.5)	0.04
Other comedication						
Oral antihistamine	707 (6.4)	113 (8.5)	0.08	635 (6.3)	62 (5.8)	0.02
Antidepressant	390 (3.5)	72 (5.4)	0.09	354 (3.5)	36 (3.3)	0.01
Antianxiety	1060 (9.6)	146 (11.0)	0.05	963 (9.6)	26 (8.4)	0.04
Oral corticosteroid	229 (2.1)	52 (3.9)	0.11	202 (2.0)	26 (2.4)	0.03
Hormone replacement therapy	30 (0.3)	6 (0.5)	0.03	28 (0.3)	1 (0.1)	0.05

Abbreviations: ACEI, angiotensin-converting enzyme inhibitor; ARB, angiotensin receptor blocker; ASMD, absolute standardized mean difference; CCI, Charlson Comorbidity Index; eGFR, estimated glomerular filtration rate; GLP-1 RAs, glucagonlike peptide-1 receptor agonists; HbA_1c_, hemoglobin A_1c_; IPTW, inverse probability of treatment weighting; NA, not applicable; SGLT2, sodium-glucose cotransporter 2; UACR, urine albumin-creatinine ratio.

SI conversion: To convert HbA_1c_ to proportion of total hemoglobin, multiply by 0.01.

^a^ Values greater than 0.1 indicate a nonnegligible difference between the 2 treatment groups.

## Results

We included a total of 10 038 patients with T2D newly receiving SGLT2 inhibitors and 1077 newly receiving GLP-1 RAs based on the study inclusion and exclusion criteria and IPTW with 5% trimming approach (eFigure in the Supplement). The SGLT2 group included 5689 men (56.7%) and 4349 women (43.3%) (mean [SD] age, 59.5 [12.1] years). The GLP-1 RA group included 587 men (54.5%) and 489 women (45.4%) (mean [SD] age, 58.5 [41.2] years). After IPTW adjustments, all baseline characteristics, including age, sex, laboratory data, ophthalmology conditions, comorbidities, and comedications, were comparable (all absolute standardized mean differences <0.1) between the SGLT2 inhibitor and GLP-1 RA groups. In the SGLT2 group, median HbA_1c_ level was 8.6% (IQR, 7.7%-9.7%), median eGFR was 89.1 mL/min/1.73 m^2^ (IQR, 71.2-108.8 mL/min/1.73 m^2^), and median UACR was 26.8 mg/g (IQR, 9.9-121.7 mg/g). In the GLP-1 RA group, median HbA_1c_ level was 8.9% (IQR, 8.0%-9.8%), median eGFR was 90.9 mL/min/1.73 m^2^ (IQR, 68.3-114.4 mL/min/1.73 m^2^), and median UACR was 41.0 mg/g (IQR, 11.8-172.2 mg/g) (Table 1).

After a mean follow-up of 3.9 years with a total of 391 DED outcomes, we observed a difference in the DED incidence between the SGLT2 inhibitor (9.0 events per 1000 patient-years) and the GLP-1 RA (11.5 events per 1000 patient-years) groups, yielding an HR of 0.78 (95% CI, 0.68-0.89) (Table 2 and Figure). However, we did not find any significant difference between the SGLT2 inhibitor and GLP-1 RA groups in regard to HbA_1c_, eGFR, and UACR changes after 1-year, 2-year, and 3-year treatments (Table 3).

**Table 2.** Risk of Dry Eye Disease for SGLT2 Inhibitors vs GLP-1 RAs

Variable	Patients	Incidence rate (95% CI) Per 1000 person-years	HR (95% CI)
Crude analysis			
SGLT2 inhibitors	11 022	8.9 (8.1-9.8)	0.86 (0.64-1.16)
GLP-1 RAs	1328	10.4 (7.9-13.6)	1 [Reference]
Main analysis (IPTW using propensity score with 5% trimming)			
SGLT2 inhibitors	10 038	9.0 (8.2-9.9)	0.78 (0.68-0.89)
GLP-1 RAs	1077	11.5 (10.5-12.6)	1 [Reference]
Sensitivity analysis			
1:1 propensity score matching			
SGLT2 inhibitors	1325	9.2 (6.9-12.2)	0.88 (0.59-1.31)
GLP-1 RAs	1325	10.4 (7.9-13.7)	1 [Reference]
IPCW accounting for informative censoring of treatment discontinuation and switch			
SGLT2 inhibitors	9976	9.0 (8.5-9.6)	0.83 (0.70-0.98)
GLP-1 RAs	1139	11.0 (9.4-12.8)	1 [Reference]
On-treatment analysis			
SGLT2 inhibitors	10 038	7.1 (6.1-8.2)	0.45 (0.37-0.54)
GLP-1 RAs	1077	15.8 (14.1-17.5)	1 [Reference]
Censored dry eye disease outcomes within 180 d			
SGLT2 inhibitors	10 038	8.3 (7.5-9.2)	0.86 (0.74-0.99)
GLP-1 RAs	1077	9.6 (8.7-10.6)	1 [Reference]
Different follow-up periods: 1 y			
SGLT2 inhibitors	10 038	7.2 (5.8-9.0)	0.61 (0.46-0.81)
GLP-1 RAs	1077	11.8 (9.9-14.1)	1 [Reference]
Different follow-up periods: 2 y			
SGLT2 inhibitors	10 038	7.3 (6.3-8.6)	0.62 (0.51-0.76)
GLP-1 RAs	1077	11.8 (10.4-13.2)	1 [Reference]
Different follow-up periods: 3 y			
SGLT2 inhibitors	10 038	7.6 (6.7-8.6)	0.64 (0.54-0.76)
GLP-1 RAs	1077	11.8 (10.6-13.1)	1 [Reference]
False outcome (incident vitreous floaters)			
SGLT2 inhibitors	10 071	1.0 (0.8-1.4)	1.31 (0.84-2.05)
GLP-1 RAs	1084	0.8 (0.6-1.2)	1 [Reference]

Abbreviations: GLP-1 RAs, glucagonlike peptide-1 receptor agonists; HR, hazard ratio; IPCW, inverse probability of censoring weighting; IPTW, inverse probability of treatment weighting; SGLT2, sodium-glucose cotransporter 2.

**Figure.**
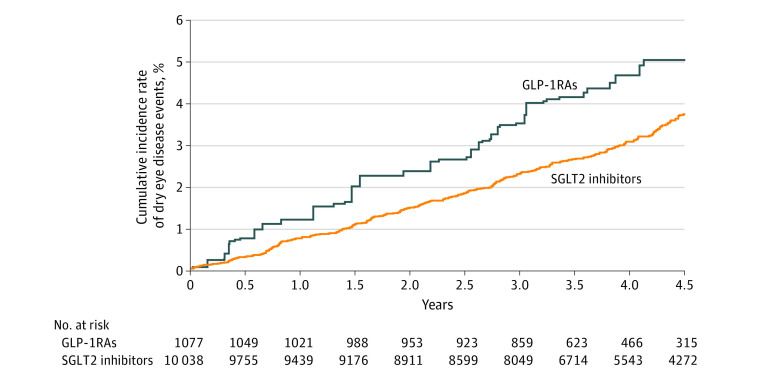
Cumulative Incidence of Dry Eye Disease Between Sodium-Glucose Cotransporter 2 (SGLT2) Inhibitor and Glucagonlike Peptide-1 Receptor Agonist (GLP-1 RA) Use After Inverse Probability of Treatment Weighting Adjustment

**Table 3.** Glycemic Control and Kidney Function Changes Posttreatment in the IPTW of Propensity Score With 5% Cohort Trimming

Variable	SGLT2 inhibitors	GLP-1 RAs	ASMD^a^
Baseline HbA_1c_, median (IQR), %	8.6 (7.7-9.7)	8.9 (8.0-9.8)	0.05
HbA_1c_, median (IQR), %			
1 y	7.8 (7.1-8.7)	7.8 (7.0-8.8)	<0.01
2 y	7.8 (7.1-8.6)	7.8 (7.0-8.8)	0.02
3 y	7.7 (7.0-8.5)	7.7 (6.9-8.7)	0.02
Baseline eGFR, median (IQR), mL/min/1.73 m^2^	89.1 (71.2-108.8)	90.9 (68.3-114.4)	0.02
eGFR, median (IQR), mL/min/1.73 m^2^			
1 y	87.6 (69.2-107.9)	88.4 (66.3-113.2)	<0.01
2 y	85.7 (67.2-105.1)	85.1 (63.4-109.6)	<0.01
3 y	84.9 (66.6-104.5)	85.1 (61.8-103.8)	0.01
Baseline UACR, median (IQR), mg/g	26.8 (9.9-121.7)	41.0 (11.8-172.2)	0.02
UACR, median (IQR), mg/g			
1 y	28.4 (10.7-119.0)	39.3 (11.9-148.0)	0.03
2 y	28.4 (10.9-124.0)	38.9 (14.1-167.0)	0.03
3 y	30.1 (11.4-129.0)	39.3 (12.8-168.0)	0.01

Abbreviations: ASMD, absolute standardized mean difference; eGFR, estimated glomerular filtration rate; GLP-1 RAs, glucagonlike peptide-1 receptor agonists; HbA_1c_, hemoglobin A_1c_; IPTW, inverse probability of treatment weighting; SGLT2, sodium-glucose cotransporter-2; UACR, urine albumin-creatinine ratio.

SI conversion: To convert HbA_1c_ to proportion of total hemoglobin, multiply by 0.01.

^a^ Values greater than 0.1 indicate a nonnegligible difference between the 2 treatment groups.

These results from the subgroup analyses suggested that the lower risks of DED from SGLT2 inhibitor use were generally similar to those from the main analysis (Table 4). However, greater reduction of DED risk was observed in men (HR, 0.62; 95% CI, 0.51-0.75), patients with eGFR levels greater than or equal to 90 mL/min/1.73 m^2^ (HR, 0.62; 95% CI, 0.51-0.77 mL/min/1.73 m^2^), and in those with UACR levels less than 30 mg/g (HR, 0.65; 95% CI, 0.54-0.78 mg/g). The results from several sensitivity analyses were also similar to those from the main analysis. For example, compared with GLP-1 RAs, the lower risks of DED associated with SGLT2 inhibitors were also observed in the 1:1 propensity score–matching analysis (HR, 0.88; 95% CI, 0.59-1.31) and on-treatment analysis (HR, 0.45; 95% CI, 0.37-0.54). The greater DED risk reductions with SGLT2 inhibitors also remained consistent for the 1-year (HR, 0.61; 95% CI, 0.46-0.81), 2-year (HR, 0.62; 95% CI, 0.51-0.76), and 3-year (HR, 0.64; 95% CI, 0.54-0.76) follow-up periods. In the analysis using the inverse probability of censoring weighting approach, we found that patients newly receiving SGLT2 inhibitors also had lower risks of DED compared with those receiving GLP-1 RAs (HR, 0.83; 95% CI, 0.70-0.98). In addition, we did not observe any significant difference in the incidence of vitreous floaters (falsification outcome) between the SGLT2 inhibitor (1.0 per 1000 person-years) and the GLP-1 RA (0.8 per 1000 person-years) groups, yielding an HR of 1.31 (95% CI, 0.84-2.05) (Table 2).

**Table 4.** Subgroup Analyses

Variable	Patients, No.	Incidence rate (95% CI) per 1000 person-years	HR (95% CI)
Age			
≥60 y			
SGLT2 inhibitor	5529	11.4 (10.1-12.8)	0.83 (0.70-0.98)
GLP-1 RA	493	13.6 (12.2-15.2)	1 [Reference]
<60 y			
SGLT2 inhibitor	4538	6.3 (5.3-7.5)	0.88 (0.69-1.12)
GLP-1 RA	556	7.2 (6.1-8.6)	1 [Reference]
Sex			
Male			
SGLT2 inhibitor	5855	6.7 (5.8-7.8)	0.62 (0.51-0.75)
GLP-1 RA	514	11.1 (9.8-12.5)	1 [Reference]
Female			
SGLT2 inhibitor	4394	11.8 (10.3-13.4)	0.93 (0.77-1.11)
GLP-1 RA	565	12.4 (10.9-14.1)	1 [Reference]
HbA_1c_ level			
≥7%			
SGLT2 inhibitor	9222	9.0 (8.1-9.9)	0.83 (0.72-0.96)
GLP-1 RA	1029	10.8 (9.8-11.9)	1 [Reference]
<7%			
SGLT2 inhibitor	824	9.5 (6.7-13.5)	0.85 (0.51-1.43)
GLP-1 RA	37	11.2 (7.7-16.2)	1 [Reference]
eGFR level			
≥90 mL/min/1.73 m^2^			
SGLT2 inhibitor	4878	7.3 (6.2-8.5)	0.62 (0.51-0.77)
GLP-1 RA	481	11.7 (10.3-13.4)	1 [Reference]
<90 mL/min/1.73 m^2^			
SGLT2 inhibitor	5206	10.9 (9.7-12.4)	0.95 (0.80-1.14)
GLP-1 RA	550	11.5 (10.1-13.1)	1 [Reference]
UACR level			
≥30 mg/g			
SGLT2 inhibitor	4721	9.0 (7.8-10.4)	0.97 (0.79-1.20)
GLP-1 RA	673	9.2 (7.9-10.6)	1 [Reference]
<30 mg/g			
SGLT2 inhibitor	5315	8.7 (7.6-10.1)	0.65 (0.54-0.78)
GLP-1 RA	408	13.5 (12.0-15.2)	1 [Reference]

Abbreviations: eGFR, estimated glomerular filtration rate; GLP-1 RA, glucagonlike peptide-1 receptor agonist; HbA_1c_, hemoglobin A_1c_; HR, hazard ratio; SGLT2, sodium-glucose cotransporter 2; UACR, urine albumin-to-creatinine ratio.

## Discussion

The findings of this large multi-institutional clinical cohort study suggest that, compared with GLP-1 RA use, SGLT2 inhibitor use was associated with a 22% lower risk of DED. Given that DED affects about one-fifth of patients with T2D and reduces the patients’ quality of life,^4,5^ our findings with the small absolute risk difference (2.5 per 1000 person-years) between SGLT2 inhibitors and GLP-1 RAs may provide an important reference for clinical decisions about prescribing different antidiabetic medications to delay or prevent DED in patients with T2D. In addition, similar changes in glycemic control and kidney function for SGLT2 inhibitors and GLP-1 RAs implied that possible mechanisms underlying the lower incidence of DED from SGLT2 inhibitor use are independent of these factors.

Clinical management of DED for most patients with T2D includes topical treatment prescribed by ophthalmologists^6^ and adequate glycemic control overseen by primary care physicians.^45^ Newer drugs, such as lifitegrast (inhibition of a specific T cell–mediated inflammatory pathway)^46^ and varenicline (nicotinic acetylcholine receptor agonist),^47^ were recently approved for treatment of DED by the US Food and Drug Administration in 2016 and 2021, respectively, but the influence of antidiabetic drugs on the incidence of DED remains unknown. In addition, as the core strategy of T2D management has been evolving from glycemic control to organ protection, the ocular therapeutic or adverse effects of novel antidiabetic drugs have been somewhat neglected.^48^ To our knowledge, this study is the first to report that SGLT2 inhibitors were associated with a decreased risk of DED compared with GLP-1 RAs. However, DED is a complicated ocular disease, which can be classified into aqueous tear deficiency, evaporative DED, or a combination of both types,^6^ so it is difficult to identify exactly the clinical roles of SGLT2 inhibitors in the pathophysiologic process of DED based on the current observational study designs. Because most types of DED lead to tear film hyperosmolarity and stimulate ocular surface inflammation in patients with T2D,^6,8^ SGLT2 inhibitors may play a role in the inflammatory process, thus blocking the vicious cycle of DED.^11,12^ Another reason why SGLT2 inhibitors may have anti-inflammatory effects on the ocular surface could be that DED has been shown to be associated with proinflammatory M1-polarized macrophages and an elevation of inflammatory cytokine levels.^8^ Sodium-glucose cotransporter 2 inhibitors have been reported to reduce M1-polarized macrophage accumulation while inducing the anti-inflammatory M2 phenotype of macrophages^11^ and to lower inflammatory cytokines by modulating the Nodlike receptor family pyrin domain-containing 3 inflammasome through ketonemia.^49^ This action could explain the greater DED risk reduction with SGLT2 inhibitors compared with GLP-1 RAs. Together with the positive findings from previous studies related to different ocular inflammatory diseases (eg, diabetic retinopathy, diabetic macular edema, and glaucoma),^15,16,17^ clinicians may also evaluate the risks for ophthalmologic conditions when selecting antidiabetic medications as intensification therapy to optimize the treatment benefits. Further research should explore the mechanisms of SGLT2 inhibitors in ocular tissues in patients with T2D.

From the results of the subgroup analyses, we found a likely greater DED risk reduction using SGLT2 inhibitors in men and in patients with better-preserved kidney function (eg, eGFR≥90 mL/min/1.73 m^2^ and UACR<30 mg/g). Sex may be a crucial risk factor for DED as supported by our observations of 1.5-fold higher incidence rates of DED in women compared with men with T2D. Women may be more likely to develop DED due to other systemic factors, such as lower androgen levels, a higher prevalence of autoimmune diseases, and higher sensitivity to pain.^50^ Similarly, patients with renal impairment or proteinuria have been shown to be at increased risk of DED due to tear hyperosmolarity and increased ocular surface inflammation.^51,52,53^ We observed 1.2-fold higher incidence rates of DED in patients both with eGFR less than 90 mL/min/1.73 m^2^ and with UACR greater than or equal to 30 mg/g compared with those with better kidney function. Because there were no significant differences in DED risks between SGLT2 inhibitor and GLP-1 RA use in women with T2D or patients with worse kidney function, the prescription of SGLT2 inhibitors for these populations at high risk of DED should be based on other clinical considerations.

Aside from chronic inflammation,^5^ hyperglycemia alone is known to be associated with superficial punctate keratopathy, trophic ulcers, persistent epithelial defects, recurrent corneal erosions, and lower values of tear secretion and tear film break-up time, which may cause DED in patients with T2D.^5^ In this study, we evaluated HbA_1c_ levels during the follow-up periods to explore possible mechanisms of DED with SGLT2 inhibitors. Because our results showed no significant difference in HbA_1c_ level changes between the SGLT2 inhibitor and GLP-1 RA groups, we hypothesized that the lower DED risks of SGLT2 inhibitors may be attributed to other ocular anti-inflammatory effects in addition to the benefits from lowered glucose levels.

### Strengths and Limitations

The strength of this study includes analysis of the largest multi-institutional database in Taiwan to evaluate the associations between DED and SGLT2 inhibitors or GLP-1 RAs in clinical practice. Also, our findings appear to support the validity of the study outcome definitions by incorporating the combination of diagnosis codes and DED medication codes.^54,55^ Third, our study introduced IPTW adjustments to make comparisons between the 2 treatment groups more homogeneous, especially by the incorporation of baseline laboratory data, such as HbA_1c_, eGFR, and UACR levels—all contributing factors for DED.^51,52,53,56^ Fourth, we consider our findings to be robust because the risk profiles of SGLT2 inhibitors vs GLP-1 RAs remained consistent after a series of sensitivity analyses (eg, different follow-up periods and falsification end point analysis).

The study has limitations. First, our results cannot be generalized to SGLT2 inhibitors other than empagliflozin, dapagliflozin, and canagliflozin and to GLP-1 RAs other than liraglutide and dulaglutide. Second, because the CGRD only includes electronic medical records from the Chang Gung Memorial Hospitals, possible differences owing to loss of follow-up or missing data cannot be ignored. Third, the much larger sample size of the SGLT2 inhibitor group may obscure greater heterogeneity within this group compared with the GLP-1 RA group in our analysis. Most of the heterogeneous factors (eg, variability of the ocular surface) are unlikely to have affected clinician decision-making on whether to prescribe SGLT2 inhibitors or GLP-1 RAs, but other factors, such as patients’ baseline diabetes severity and adherence to drugs, may have played a greater role. However, we have included patients’ baseline HbA_1c_ level to address the severity of diabetes through propensity score–matching methods. We also applied the inverse probability of censoring weighting approach to address informative censoring owing to discontinuation or switching of drugs, and the results remained consistent with the main analysis. Nevertheless, some residual confounding effects may not have been fully eliminated by these methods. Fourth, the validity of the DED diagnoses in the database has not been verified. To improve the outcome validity, we have therefore defined the outcome by diagnosis identified by *ICD-10-CM* codes along with prescription records of medications for treatment of DED, including artificial tears and topical cyclosporine. In accordance with the reimbursement guidelines of Taiwan's National Health Insurance, artificial tears should be prescribed only if the basal Schirmer test shows less than 5 mm of wetting or when patients have keratopathy caused by DED; topical cyclosporine should be prescribed only when patients show less than 5 mm of wetting in the basal Schirmer test, have a tear film break-up time less than 5 seconds, and DED severity is higher than grade 3. Clinical experts from Taiwan’s National Health Insurance Administration regularly review the prescriptions, confirming the DED diagnosis. A validation study for DED in the Taiwan database is lacking; however, misclassifications of outcome occurrence may be nondifferential between groups. Although we may therefore have underestimated the differences in the risks between the 2 groups because misclassification of DED causes bias toward the null, the conclusion remains sound. In addition, owing to the nature of retrospective study design, we did not evaluate clinical types in patients developing DED. Additional studies with prespecified clinical and instrumental diagnostic assessment are suggested to investigate the role of SGLT2 inhibitors in DED incidence.

## Conclusions

In this cohort study, the use of SGLT2 inhibitors in patients with T2D was associated with a 22% lower DED incidence compared with GLP-1 RA use. These findings provide a basis for future research into possible protective associations between SGLT2 inhibitor use and DED.

## Notes

Supplement. **eFigure.** Cohort Selection **eTable 1.** Taiwan’s National Health Insurance Reimbursement Guidelines for Artificial Tears and Topical Cyclosporine **eTable 2.** Diagnosis Codes for Study Outcome and Comorbidities **eTable 3.** Individual Comedication Drugs
