# Supplementary material for: Comparison of Sodium-Glucose Cotransporter 2 Inhibitors vs Glucagonlike Peptide-1 Receptor Agonists and Incidence of Dry Eye Disease in Patients With Type 2 Diabetes in Taiwan
Source: JAMA Netw Open. 2022 Sep 22;5(9):e2232584. doi: 10.1001/jamanetworkopen.2022.32584 (PMC9500553; doi:10.1001/jamanetworkopen.2022.32584)
Supplement: Supplement. — eFigure. Cohort Selection eTable 1. Taiwan’s National Health Insurance Reimbursement Guidelines for Artificial Tears and Topical Cyclosporine eTable 2. Diagnosis Codes for Study Outcome and Comorbidities eTable 3. Individual Comedication Drugs [file jamanetwopen-e2232584-s001.pdf]

Supplementary Online Content

Su YC, Hung JH, Chang KC, et al. Comparison of sodium glucose cotransporter 2 inhibitors vs glucagonlike peptide-1 receptor agonists and incidence of dry eye disease in patients with type 2 diabetes in Taiwan. *JAMA Netw Open*. 2022;5(9):e2232584. doi:10.1001/jamanetworkopen.2022.32584

eFigure. Cohort Selection

eTable 1. Taiwan's National Health Insurance Reimbursement Guidelines for Artificial Tears and Topical Cyclosporine

eTable 2. Diagnosis Codes for Study Outcome and Comorbidities

eTable 3. Individual Comedication Drugs

This supplementary material has been provided by the authors to give readers additional information about their work.

© 2022 Su YC et al. *JAMA Network Open.*

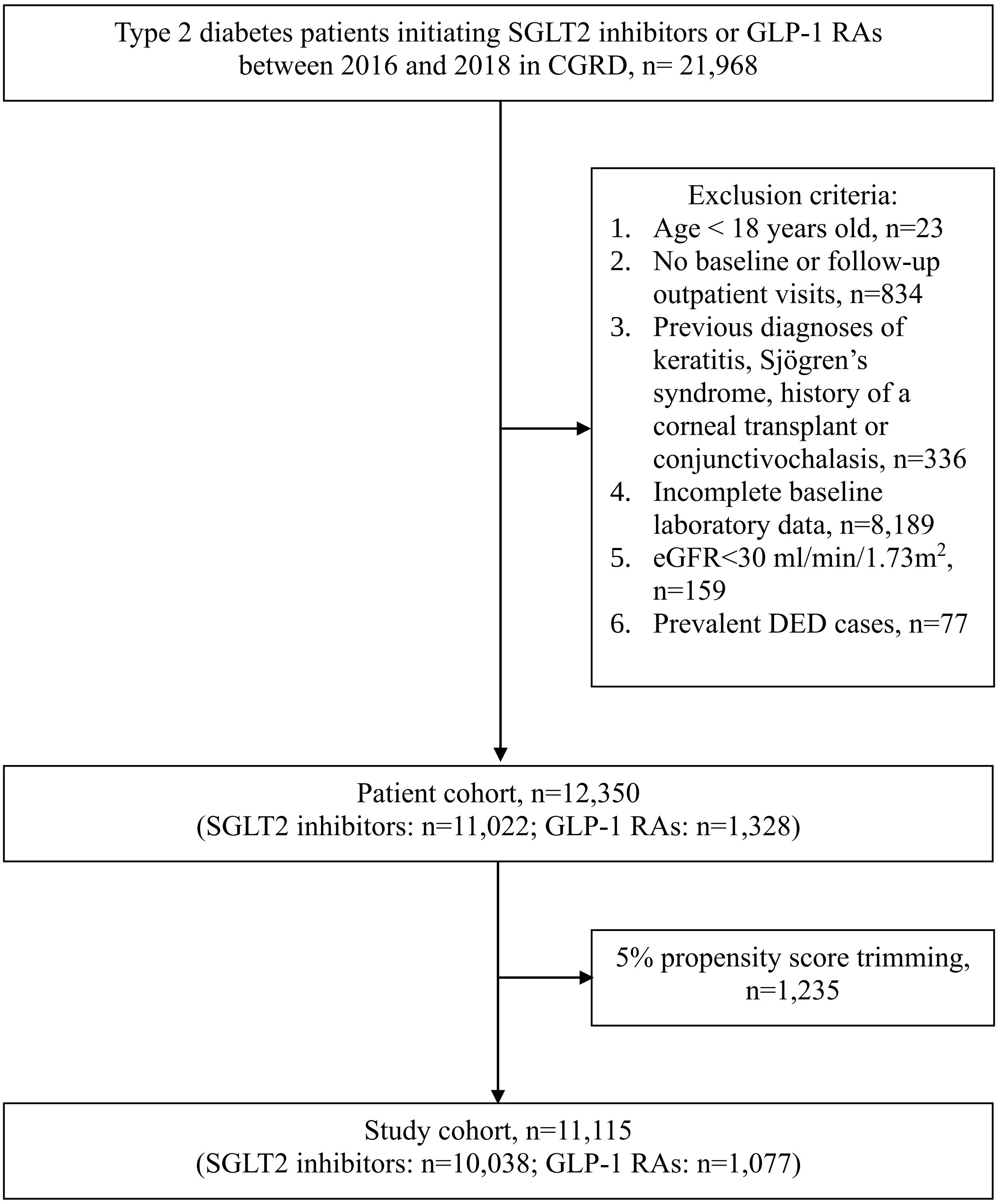

eFigure. Cohort selection.

Abbreviations: eGFR: estimated glomerular filtration rate, GLP-1 RA: glucagon-like peptide-1 receptor agonist, IPTW: inverse probability of treatment weighting, SGLT2: sodium-glucose co-transporter 2.

© 2022 Su YC et al. *JAMA Network Open.*

eTable 1. Taiwan's National Health Insurance reimbursement guidelines for artificial tears and topical cyclosporine

Medications	Reimbursement guidelines
Artificial tears	1. Basal Schirmer test shows less than 5 mm of wetting,<br>or<br>2. Patients have keratopathy caused by dry eye disease, exposure keratopathy, or other related keratopathies.
Topical cyclosporin	1. Dry eye disease severity is over grade 3,<br>and<br>2. Basal Schirmer test shows less than 5 mm of wetting,<br>and<br>3. Tear film break-up time is less than or equal to 5 seconds,<br>and<br>4. Corneal surface images under fluorescein stain show punctate epithelial erosions, injected conjunctiva with filamentary keratitis, corneal ulcer, symblepharon, or keratinization of ocular surface epithelium,<br>and<br>5. Patient tried topical anti-inflammatory agents or punctal occlusion or artificial tears but had limited treatment response.

© 2022 Su YC et al. *JAMA Network Open.*

**eTable 2.** Diagnosis codes for study outcome and comorbidities		
Diseases	ICD-9-CM codes	ICD-10-CM codes
**Study outcome**		
Dry eye disease	37515	H0412
**False outcome**		
Vitreous floaters	37924	H4339
**Exclusion criteria**		
Keratitis	370	H16
Sjögren's syndrome	7102	M350
History of a corneal transplant	V42.5	Z947
Conjunctivochalasis	37281	H1182
**Co-morbidities**		
Diabetic complications		
Diabetic retinopathy	2505, 3620	E113
Diabetic neuropathy	2506	E114
Diabetic nephropathy	2504	E112
Ophthalmological conditions		
Myopia	36021, 3671	H442, H521
Presbyopia	3674	H524
Glaucoma	365	H40, H42
Uveitis	36012, 3630, 3631, 36320, 36321, 36322, 3640, 3641, 3642, 3643	H20, H300, H301, H302, H308, H309, H4411
Blepharitis	3730	H010
Conjunctivitis	3720, 3721, 3722, 3723	H100, H101, H102, H103, H104, H105, H1089, H109
Pterygium / Pinguecula	3724, 3725	H1081, H110
Cardiovascular co-morbidities		
Coronary heart disease	410, 411, 412, 413, 414	I20, I21, I22, I23, I24, I25
Ischemic stroke	433, 434	I63
Peripheral artery disease	440	I70, I73
Heart failure	428	I50
Hypertension	401, 402, 403, 404, 405	I10, I11, I12, I13, I15, I16
Atrial fibrillation	42731	I48
Dyslipidemia	272	E78

© 2022 Su YC et al. *JAMA Network Open.*

Other co-morbidities		
Asthma	493	J45
Hypothyroidism / Hyperthyroidism	242, 244	E03, E05
Liver disease	456, 571, 572	I85, K70, K71, K72, K73, K74, K75, K76, K77
Benign prostatic hyperplasia	600	N40
Chronic obstructive pulmonary disease	491, 492, 496	J44
Arthritis	7162, 7163, 7165, 7166	M13
Gout	274	M10, M1A
Rheumatoid diseases	4465, 7100, 7101, 7103, 7104, 7140, 7141, 7142, 7148, 725	M051, M052-M059, M316, M321, M328, M329, M3303, M3313, M332, M3390, M3393, M340, M341, M349, M353
Migraine	346	G43
Sleep apnea	32723	G473
Depression	2962, 2963	F32, F33
Schizophrenia	295	F20
Rosacea	6953	L71
ICD-9-CM: International Classification of Diseases, Ninth Revision, Clinical Modification, ICD-10-CM: International Classification of Diseases, Tenth Revision, Clinical Modification		

© 2022 Su YC et al. *JAMA Network Open.*

**eTable 3.** Individual comedication drugs	
Drug class	ATC code
Cardiovascular co-medication	
Anti-platelet agents	B01AC04, B01AC07, B01AC24, N02BA01
Beta-blockers	C07AA05, C07AB02, C07AB03, C07AB07, C07AB12, C07AG02
ACEI or ARBs	C09AA01, C09AA02, C09AA05, C09CA01, C09CA04, C09CA06, C09CA07, C09CA09, C09CA08, C09AA09, C09DB01
Calcium channel blockers	C08CA01, C08CA02, C08CA05, C08CA13, C08DB01, C08DA01
Diuretics	S01EC01, C03EA01, C03AA, C03CA01, C03CA02, C03DA01, C03DA04, C02LA51, C03BA11
Statin	C10AA01, C10AA04, C10AA05, C10AA08, C10AA07
Fibrate	C10AB04, C10AB05
Diabetes co-medication	
Sulfonylurea	A10BB01, A10BB07, A10BB09, A10BB12
Dipeptidyl peptidase-4 inhibitors	A10BH01, A10BH02, A10BH03, A10BH04, A10BH05
Glinides	A10BX02, A10BX03, A10BX08
Thiazolidinediones	A10BG03
Insulins	A10AB01, A10AC01, A10AD01, A10AD04
Other co-medication	
Oral antihistamines	R06
Antidepressants	N06A
Antianxiety medications	N05B
Oral steroids	H02
Hormone replacement therapy	G03C, G03F, G03H
ACEI: Angiotensin converting enzyme inhibitor, ARB: Angiotensin receptor blocker	

© 2022 Su YC et al. *JAMA Network Open.*
